# Establishment of an immortalised human ovarian surface epithelial cell line without chromosomal instability

**DOI:** 10.1038/sj.bjc.6602662

**Published:** 2005-06-14

**Authors:** T Maeda, H Tashiro, H Katabuchi, M Begum, H Ohtake, T Kiyono, H Okamura

**Affiliations:** 1 Department of Reproductive Medicine and Surgery, Faculty of Medical and Pharmaceutical Science, Kumamoto University, Honjo 1-1-1, Kumamoto-City, Kumamoto 860-8556, Japan; 2 Department of Gynecology, Faculty of Medical and Pharmaceutical Science, Kumamoto University, Honjo 1-1-1, Kumamoto-City, Kumamoto 860-8556, Japan; 3 Virology Devision, National Cancer Center Research Institute, 5-1-1 Tsukiji, Chuo-ku, Tokyo 104-0045, Japan

**Keywords:** human, ovarian surface epithelium, immortalisation, human papillomavirus, hTERT

## Abstract

Epithelial ovarian carcinoma is thought to derive from ovarian surface epithelium (OSE). The black box of the early molecular changes in ovarian carcinogenesis is being interpreted by the development of experimental systems employing immortalised human OSE cells. However, the existing cell lines of the OSE cells have limited utility due to chromosomal instability. Our goal was to establish new immortalised human OSE cells that retain the original characteristics of the primary cells without chromosomal alterations. Using primary human OSE cells obtained from a postmenopausal patient with endometrial cancer, five cell lines (‘HOSE1’ lines) were newly established by infection with retroviral expression vectors containing type 16 human papillomavirus (HPV-16) E6, E7, a variant E6 (E6Δ151), and Bmi1 polycomb gene, in combination with telomerase reverse transcriptase (hTERT). Consequently, five HOSE1s cell lines, HOSE1s-E6/hTERT, -E7/hTERT, -E6/E7/hTERT, -E6Δ151/E7/hTERT, and -E6Δ151/Bmi1/hTERT, grew beyond the population doubling number of 200. These cell lines, except for HOSE1-E6/hTERT, essentially showed the original features of the primary human OSE cells. Of them, HOSE1-E7/hTERT preserved diploidy in a kariotype analysis, and did not show transformed phenotypes in anchorage-independent growth and tumour formation. Thus, HOSE1-E7/hTERT may provide a novel model system with which to investigate the mechanisms of early molecular changes.

Most epithelial ovarian carcinomas are considered to arise from ovarian surface epithelium (OSE), a single continuous layer of flat-to-cuboidal mesothelial cells covering the ovary (Okamura and Katabuchi, 2005). In epithelial ovarian carcinoma, numerous studies have revealed different patterns of genetic change. These include activation of oncogenes (K-ras, HER-2, Src, c-myc, AKT2, PIK3CA and STK/BTAK) through amplification, translocation, or mutation (Slamon *et al*, 1989; Enomoto *et al*, 1991; Tashiro *et al*, 1992; Bellacosa *et al*, 1995; Zhou *et al*, 1998; Shayesteh *et al*, 1999; Wiener *et al*, 2003), as well as inactivation of tumour suppressor genes (p53, PTEN, WT1, BRCA 1, NOEY2 and WWOX) through homozygous deletion, double mutations, or a combination of mutation and loss of heterozygosity or promotor methylation (Fujita *et al*, 1995; Obata *et al*, 1998; Yu *et al*, 1999; Schorge *et al*, 2000; Paige *et al*, 2001; McCoy *et al*, 2003). Although such genetic changes have long been identified using ovarian carcinoma tissues or cell lines, the early molecular changes in ovarian carcinogenesis have not been fully clarified. In addition, adequate experimental systems and suitable animal models are lacking due to spontaneous epithelial ovarian tumours being extremely rare in nonprimate mammals, including mice. During the last decade, the isolation, culture, and immortalisation of human OSE cells was developed (Maines-Bandiera *et al*, 1992; Tsao *et al*, 1995; Nitta *et al*, 2001). Using the experimental system of Auersperg *et al* (1999), E-cadherin expression was recently addressed in human ovarian carcinogenesis (Ong *et al*, 2000). Furthermore, in the OSE cells, autocrine HGF-Met and LH-LH/hCG receptor loops have been shown to be involved in early carcinogenesis (Wong *et al*, 2001; Tashiro *et al*, 2003). Although the system using the immortalised human OSE cell lines would serve as relevant models, the utility of the existing cell lines is limited by their accumulation of chromosomal instability. The establishment of new cell lines without such genetic instability is required to advance further research of carcinogenesis.

The proliferation potential of epithelial cells is determined by three independent stages: mortality stage 0 (M0) Foster *et al* reported (Foster and Galloway, 1996; Foster *et al*, 1998), mortality stage 1 (M1) and mortality stage 2 (M2), Shay *et al* described (Shay *et al*, 1991; Wright and Shay, 1992). Epithelial cells appear to senesce in initial growth arrest, M0, which is controlled by the p16/Rb pathway. Following progressive shortening of telomere length in cell divisions, the next growth arrest, M1, is induced by telomere shortening and activation of p53/p21 pathway. When the p16/Rb and p53 functions are inactivated, the cells overcome M0 and M1. However, even after epithelial cells have bypassed these stages, telomere shortening continues until the cells reach a crisis, M2, which is characterized by marked chromosomal instability and chromosome fusion (Zakian, 1989; Blackburn, 1991). Most of cells die in the crisis, but the remaining small number of cells acquires telomerase activity and prevents the crisis.

Primary human OSE cells have limited proliferative potential and fall into early senescence within 2-8 passages *in vitro*. Until now, using human OSE cells, two kinds of immortalised cell lines were established by introduction of SV40 large T antigen (LT) (Maines-Bandiera *et al*, 1992; Nitta *et al*, 2001) or by human papillomavirus (HPV) E6 and E7 (E6/E7) transduction (Tsao *et al*, 1995). The OSE cells transduced by SV40 LT and by E6/E7 had chromosomal alterations ranging in number from 50 to 69 (Nitta *et al*, 2001) and 43 to 88 (Tsao *et al*, 1995), respectively. These OSE cells were not thought to have active telomerase, and consequently resulted in chromosomal alterations. Hence, we postulated that inhibition of p16/Rb and/or p53 pathways in combination with activation of telomerase may induce immortalisation, while abolishing chromosomal instability. Our goal was to establish immortalised human OSE cell lines without chromosomal instability, by transducing E6 or a variant E6 with a mutation of PDZ domain-binding site for the inhibition of p53, E7 for the inhibition of RB, and polycomb family transcriptional repressor Bmi1 for inhibition of p16 (Jacobs *et al*, 1999), in combination with human telomerase reverse transcriptase (hTERT) for the activation of telomerase.

## MATERIALS AND METHODS

### Isolation of human OSE and primary cell culture

Both ovaries were obtained from a postmenopausal patient undergoing abdominal total hysterectomy and bilateral salpingo-oophorectomy for endometrial cancer (International Federation of Gynecology and Obstetrics: stage IIIc, TMN classification: pT1bN1M0, peritoneal washing smear: negative) at Kumamoto University Hospital, Japan. The Ethics Committee of Kumamoto University approved this study and the subject gave her informed consent for participation. The ovaries were grossly normal and no pathological lesions were observed by subsequent histological examination. After collagenase digestion under aseptic conditions, the OSE cells were scraped with a surgical blade as described previously (Nakamura *et al*, 1994). The viability of these cells was more than 95%, as shown by the trypan blue dye exclusion test. Subcultured OSE cells were cultured in Dulbecco's modified Eagle's medium (DMEM)/F12 (1 : 1 mixture) (Invitrogen, NY, USA) supplemented with 10% fetal bovine serum (FBS; Thermo Trace Ltd, Melbourne, Australia), 100 U ml^−1^ penicillin G (P), and 100 *μ*g ml^−1^ streptomycin (S) in a humidified 5% CO_2_ incubator at 37°C. Upon reaching confluency, cells were detached from the dish with 0.125% trypsin and 0.11% ethylenediamine tetraacetic acid (EDTA) and split in a 1 : 2 ratio in a new culture dish.

### Vector construction and retroviral transduction of E6, variant E6, E7, or Bmi1 as well as hTERT

pCMSCVpuro comprises the CMV/LTR fusion promotor, the packaging signal Psi+, and the multicloning sequence from pCLXSN (Imgenex Corp.,. San Diego, CA, USA) followed by the PGK-puro cassette and the 3′ long terminal repeat of murine embryonic stem cell virus from pMSCVpuro (Clontech, Palo Alto, CA, USA). The Gateway system (Invitrogen, Carlsbad, CA, USA) was used for subcloning genes into retroviral vectors. The destination vectors, pCLXSN-DEST and pDEST-CMSCVpuro, were constructed by inserting a modified cassette containing attR sites and ccdB (Invitrogen) between the *Xho*I and *Bam*HI sites of pCLXSN, and between the *Eco*RI and *Bgl*II sites of pCMSCVpuro, respectively. Cloning of the full-length hTERT cDNA and human Bmi-1 cDNA has been described previously (Okamoto *et al*, 2002). After cloning segments of HPV16 E6 (16E6), E7 (16E7), and a deletion mutant of HPV E6, 16E6SD-d151 (E6Δ151) (Kiyono *et al*, 1997), these segments were recombined into the retroviral vectors by the LR reaction (Invitrogen) to generate pCMSCVpuro-hTERT, pCLXSN-16E6, pCLXSN-16E6Δ151, and pCLXSN-16E7. pCLXSN-16E6E7 was constructed by inserting an *Eco*RI-*Bam*HI segment containing HPV16 E6 and E7 between the *Eco*RI and *Bgl*II sites of pCLXSN. Production of recombinant retroviruses was as previously described (Naviaux *et al*, 1996). Briefly, retroviral vector and packaging construct, pCL-10A1, were cotransduced into 293T cells using TransIT-293 (Mirus Co., Madison, WI, USA) according to the manufacturer's instructions. The culture fluid was harvested at 48 to 72 h post-transduction. The titer of the recombinant viruses was greater than 1 × 10^5^ drug-resistant colony-forming units ml^−1^ on Hela cells. A 1 ml aliquot of the culture fluid in the presence of polybrene (4 *μ*g ml^−1^) was added in 24-well dishes on which the cells were seeded. Following inoculation with viruses, cells were grown without drug selection as mock-infected cells stopped growing within 2 weeks. To achieve combinations of retroviral infections, cells were infected with LXSN-16E6, LXSN-16E7, LXSN-16E6E7, LXSN-hBmi1, or LXSN alone, at passage 2, and subsequently reinfected with MSCV-hTERT at passage 3. Alternatively, the cells infected with either LXSN-16E7 or LXSN-hBmi1 and MSCV-hTERT were additionally infected with LXSN -16E6Δ151 at passage 5.

### Cell culture

The transduced OSE cells were maintained in DMEM/F12 (1 : 1) supplemented with 10% FBS, 100 U ml^−1^ penicillin G, and 100 *μ*g ml^−1^ streptomycin in an atmosphere of 5% CO_2_ at 37°C. When confluent, transduced cells were trypsinised and subcultured in a split ratio of 1 : 4–16. Cells were defined to be immortalised when the population doubling level of the transduced cells surpassed 100 population doubling levels (Kyo *et al*, 2003). Cells were seeded into 24-well plates and each well counted daily. Growth curves were constructed, and doubling times were estimated. The human ovarian cancer cell line, NIH;OVCAR-3 (OVCAR-3), was obtained from Cell Resource Center for Biomedical Research, Tohoku University, Japan, and cultured under the same conditions as the HOSE1s cells.

### TRAP assay

In each of the cell lines, telomerase activity was examined by the telomeric repeat amplification protocol (TRAP) assay using the TRAPeze kit (Intergen, Burlington, MA, USA) according to the manufacturer's instructions.

### Western blotting

A 20 *μ*g protein sample from whole-cell extracts was separated by SDS–PAGE and blots were prepared on Immobilon-P membrane (Millipore, Billerica, MA, USA). The following antibodies were used as probes: for p53 and p21 proteins, clones DO-1 and EA-10, respectively (Oncogene Science, Cambridge, MA, USA); for Rb and p16 proteins, clones G3-245 and G175-405, respectively (PharMingen, San Diego, CA, USA); for Bmi1 protein (produced by Kiyono *et al*, unpublished). Blots were then probed with horseradish-peroxidase-conjugated goat anti-mouse IgG (Jackson Immuno Research, West Grove, PA, USA) and visualised using the chemiluminescence system (Amersham, Biosciences, Piscataway, USA).

### Reverse transcriptase–polymerase chain reaction (RT–PCR)

Total RNA was prepared from the cultured cells using Trizol reagent, as described by the manufacturer. cDNA was synthesised from 5 *μ*g of total RNA using Oligo dT_12−18_ Primers (Invitrogen, CA, USA) as the primer and Superscript II Rnase HReverse Transcriptase (Invitrogen). Amplification of E-cadherin was performed for 30 cycles (denaturation for 30 s at 94°C, annealing for 40 s at 57°C and extension for 30 s at 72°C) in a standard 50 *μ*l PCR mixture. The reaction mixture contained 1 *μ*l cDNA from the RT reaction, 0.2 *μ*M of the first pair of upstream and downstream primers, 5 *μ*l 10 × PCR buffer (Invitrogen), 0.2 mM dNTP (Invitrogen), 1.5 mM MgCl_2_ (Invitrogen), and 5 U *Taq* DNA polymerase (Invitrogen). The sequences of the RT–PCR primers were 5′-GAGGAGAGCGGTGGTCAAAG-3′ (sense) and 5′-GTTCAGGGAGCTCAGACTAG-3′ (antisense) for E-cadherin to produce a 351 bp PCR product (Nishida *et al*, 2003). The primer sequences for human glyceraldehyde-3-phosphate dehydrogenase (GAPDH) as internal control were 5′-GAA GGT GAA GGT CGG AGT-3′ (sense) and 5′-GAA GAT GGT GAT GGG ATT TC-3′ (antisense) to produce a 226 bp PCR product. Polymerase chain reaction was carried out in a Thermal Cycler (PC 701; Astec, Fukuoka, Japan). The amplified PCR products were analysed by electrophoresis through 2.0% agarose gel, visualised by ethidium bromide staining, and photographed under ultraviolet illumination. The 100 bp DNA ladder (BioLabs, Beverly, MA, USA) was used for determining the molecular size of amplified products. OVCAR-3 was employed as a positive control. As a negative control, distilled water was used in place of cDNA in all the reactions.

### Immunocytochemistry

HOSE1s cells were cultured on Lab Tech Chamber slides (Nalge Nunc International, Rochester, NY, USA) for 24 h. They were fixed in 95% ethanol for 10 min at room temperature and washed with PBS. Endogenous peroxidase activity was blocked with 0.03% hydrogen peroxide in methanol for 5 min. The cultured chamber slides were stained with the following mouse monoclonal antibodies: pan-cytokeratine (1 : 50 dilution, DAKO, Carpinterio, CA, USA), vimentin (1 : 10 dilution, DAKO), epithelial membrane antigen (EMA) antibody (1 : 200 dilution, DAKO), CA125 (1 : 100 dilution, DAKO), or collagen IV (1 : 100 dilution, Sigma-Aldrich, St Louis, MO, USA) for 30 min at room temperature. Staining reactions were performed using the indirect method using an ENVISION+ (DAKO). Peroxidase activity was visualised with 3,3′-diaminobenzidine (Sigma-Aldrich) as a substrate in Tris-HCl buffer (0.5 mg ml^−1^ pH 7.6) containing 0.01% H_2_O_2_. Nuclear staining was performed with Mayer's haematoxylin.

### CA125 levels in culture medium

Medium was changed every 2 days and was taken from cultures with a cell density varying between 0.6–1.7 × 10^6^ cells ml^−1^ growing in 6 cm dishes. The CA125 in the supernatant was measured by electrochemiluminescence immunoassay using Elecsys CA 125 (Roche Diagnostics K.K., Tokyo, Japan).

### Karyotype analysis

Karyotype analyses were performed at passage 16 and passage 41 for each cell line. Routine karyotypic analysis was performed using preparations stained with 5% Giemsa solution. In order to identify possible rearrangements, chromosomes of metaphases were G-banded. For each cell line, more than 50 cells were scored for their chromosome number. Chromosomal analysis of the most stable line at passage 41 was added at 60 passage.

### Cell proliferation assay (anchorage-dependent growth)

HOSE1s cells were placed in separate 96-well microtitre plates at a density of 2 × 10^3^ cells well^−1^ and allowed to grow for 24 h in DMEM with 10% FBS. The Biotrak Cell Proliferation ELISA System Version 2 (Amersham Pharmacia Biotech, Uppsala, Sweden) was used for the cell proliferation assay. Briefly, cells were incubated in 5-bromo-2′-deoxyuridine (BrdU) labelling solution. Following cell fixation and DNA denaturation, specimens were incubated in peroxidase-labelled anti-BdrU. Subsequently, 3,3′,5,5′-tetramethylbenzidine was added as a substrate to detect immune complexes. Absorbance was calculated from the absorbance at 450 nm measured using a microtitre plate reader.

### Assay for colony formation in soft agar (anchorage-independent growth)

Colony formation in semisolid agar was assayed by suspending 4 × 10^3^ cells in 2 ml of DMEM/F12/10% FBS with 0.33% agarose (Sigma-Aldrich) and placing this suspension on top of 2 ml of solidified 0.5% agarose in the above medium. Six cultures for each cell type were maintained for 21 days at 37°C in a 5% CO_2_ atmosphere with fresh medium changed every 1 week. Colonies larger than 100 *μ*m in diameter were counted after a lapse of 3 weeks. The experiments were repeated three times.

### Assay for tumour formation in athymic *nu/nu* mice

Female athymic BALB/cA *nu/nu* mice, aged 4 weeks and weighting 15–18 g, were obtained from CLEA Japan (Tokyo, Japan). Mice were maintained in a clean box at 22±2°C and 50–70% humidity on a 12 h light–dark cycle. Gamma-ray-irradiated solid food was provided by Oriental Yeast (Tokyo, Japan), and drinking water was acidified with HCl to a pH of 2.5–3.0. All procedures in this assay were approved by the Center for Animal Resources and Development of Graduate School of Medical Science, Kumamoto University.

Cells of E7/hTERT and OVCAR-3 were harvested with trypsin: EDTA, and the pellet of cells obtained by centrifugation was suspended in growth medium (3.6 × 10^7^ cells ml^−1^). Aliquots (0.1 ml) were injected subcutaneously into the subscapular areas of athymic BALB/c *nu/nu* mice and observed for tumorigenic growth every week for a 4-month period.

### Statistical analysis

The Mann–Whiteney *U*-test was used to assess the differences in the proliferation assay. Differences were considered to be significant at *P*<0.05. This analysis was made using the StatView system (Abacus, Berkeley, CA, USA).

## RESULTS

### Telomerase and cell cycle regulator in primary human OSE and transduced cells

**Figure 1:**
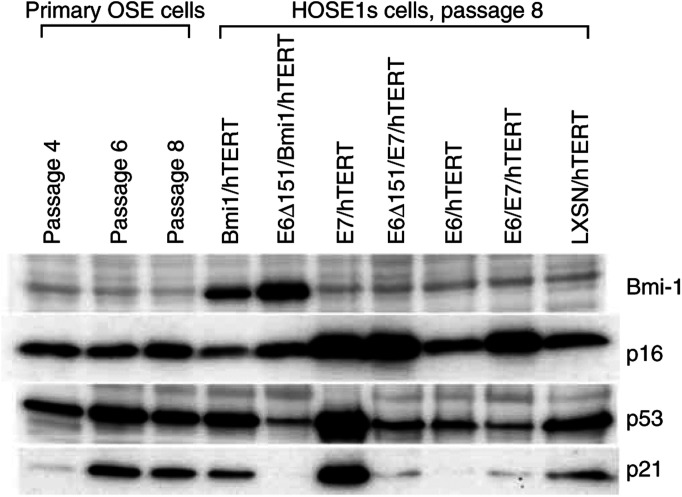
Western blot analysis of Bmi1, p16, p53, and p21 proteins in primary human OSE cells and the immortalised HOSE1s cells. The left three lanes indicated primary human OSEs cells at passages 4, 6, and 8. The other seven lanes showed expression in the immortalised HOSE1s cells at passage 8. LXSN/hTERT was HOSE1 cells transduced with hTERT alone.

All transduced cells showed telomerase activity in a TRAP assay at passage 7 (data not shown). In primary human OSE cells, p16 protein levels were quite high as early as passage 4, and levels of p53 and p21 proteins increased through passage 4–6 (Figure 1). Expression of p53 and p21 proteins in all seven transduced HOSE1 cell groups were inhibited by harbouring E6 or E6Δ151 (HOSE1s-E6/hTERT, -E6/E7/hTERT, -E6Δ151/E7/hTERT, and -E6Δ151/Bmi1/hTERT), while they were increased by E7 alone (HOSE1-E7/hTERT) when compared with those of primary cells (Figure 1). It could be supported by the fact that blockage of Rb pathway enhances p53 pathway via induction of p14ARF (Bates *et al*, 1998). The expression of p16 protein was slightly suppressed by Bmi1 alone (HOSE1-Bmi1/hTERT), and was elevated in cells expressing E7 (HOSE1s-E7/hTERT, -E6/E7/hTERT and -E6Δ151/E7/hTERT), as E7 allowed high p16 levels through inactivation of Rb (Figure 1). Our data were consistent with previous studies that immortalised cells by inactivating Rb pathway introducing E7 highly expressed p53, p21, and p16 (Foster and Galloway, 1996; Kiyono *et al*, 1998; Jarrard *et al*, 1999; Mori *et al*, 2005).

### Long-term culture and immortalisation by transduction

**Figure 2:**
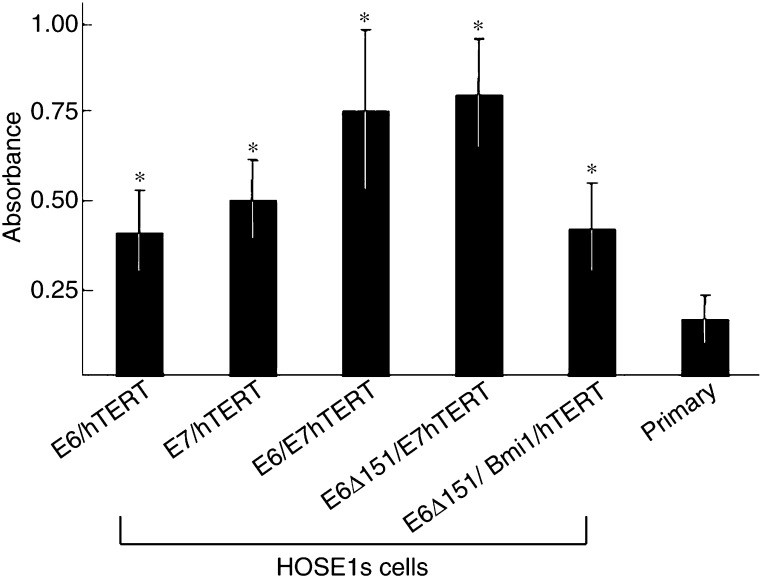
Cell proliferation assay of the immortalised HOSE1s cells and primary OSE cells. Data represented the absorbance of each cell type. Individual values were compared using Mann–Whitney's *U*-test and were significantly different from primary OSE cells (^*^; *P*<0.05).

The two cell lines HOSE1s-hTERT and -Bmi1/hTERT fell into senescence within passages 8–10, whereas the cells of HOSE1s-E6/hTERT, -E7/hTERT, -E6/E7/hTERT, -E6Δ151/E7/hTERT, and -E6Δ151/Bmi1/hTERT grew beyond a population doubling number of 100. Consequently, they were defined as immortalised cell lines. Moreover, these five cell lines are now growing with a population doubling number greater than 200. The doubling time (DT) of the HOSE1s cells was 23.4–36.9 h (Table 1). Furthermore, in the anchorage-dependent proliferation assay, cell proliferation of HOSE1s cells was significantly higher than that of primary human OSE cells (*P*<0.05) (Figure 2). The three cell lines, HOSE1s-E6/E7/hTERT, -E6Δ151/E7/hTERT, and -E6Δ151/Bmi1/hTERT, inhibited both p16/Rb and p53, and showed shorter DT and possessed higher proliferative ability than -E6/hTERT and -E7/hTERT cells (Figure 2 and Table 1).

### Karyotype analysis

**Figure 3:**
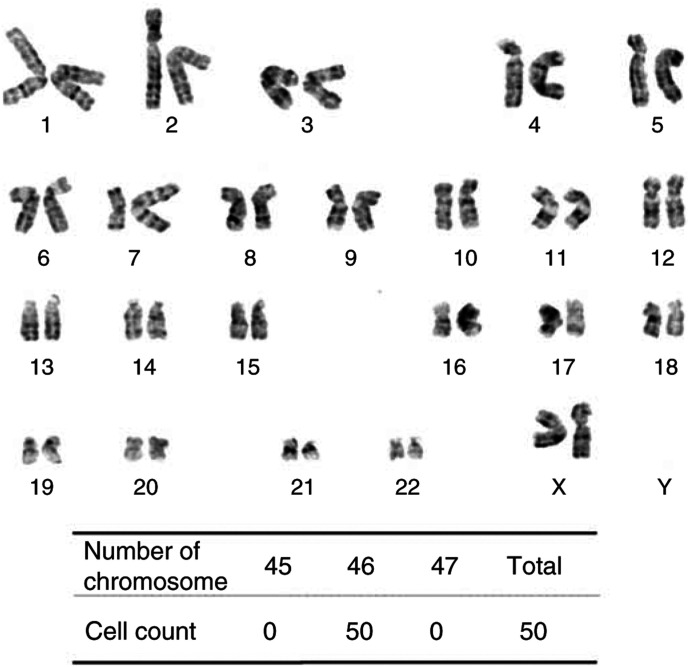
Karyotype of HOSE1-E7/hTERT cells. The chromosomal number was diploid after long–term culture (passage number 41; population doubling 142).

Although HOSE1s-E6/hTERT, -E6/E7/hTERT, -E6Δ151/E7/hTERT, and -E6Δ151/Bmi1/hTERT exhibited almost normal chromosomal number with small deviation ranging between 42 and 48 in early passage (passage 16), they did show chromosomal alteration varying from 43 to 96 after immortalisation (passage 41) (Table 2A). HOSE1-E7/hTERT alone revealed normal diploidy in both passages (Figure 3 and Table 2B). In late passage (passage 41), the G-banded karyotype of this cell line was normal in five of 12 cells, 46, XX, showed minor structural alteration including 46, XX, t(7;7)(p10;p10) in five of 12 cells, and showed 46, idem, -X, +i(X)(p10), add(1)(p36), add(10)(p11) in two of 12 cells (Table 2B). Futhermore, karyotype of the HOSE1-E7/hTERT cells at passage 60 was additionally analysed. All 50 cells revealed also diploidy and the G-banded karyotype of this was 46, XX in three of 13 cells and showed a minor structural alteration indicated 46, XX, t(7;7)(p10;p10) in 10 of 13 cells.

### Morphological characterisation

**Figure 4:**
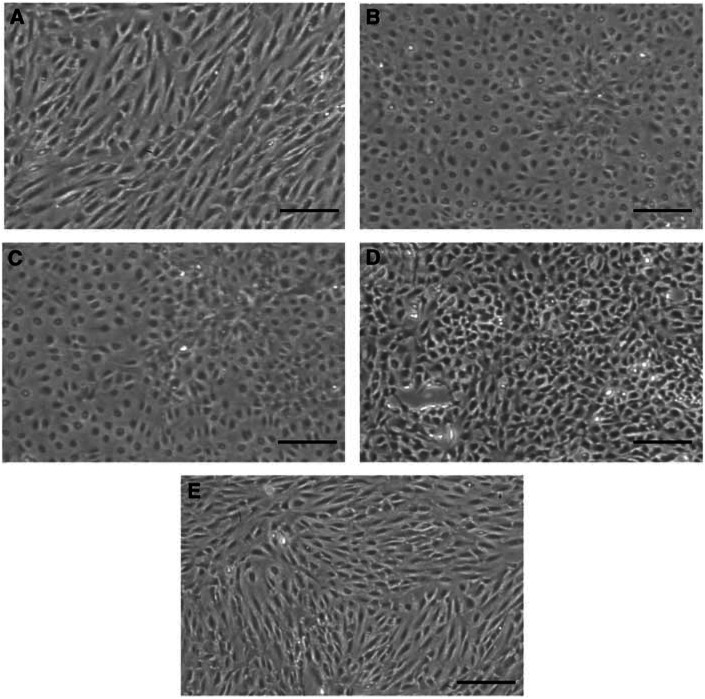
Morphological phenotype of immortalised HOSE1-E6/hTERT, -E7/hTERT, -E6/E7/hTERT, -E6Δ151/E7/hTERT and -E6Δ151/Bmi1/hTERT. Passage number of all HOSE1 cells was 40. The morphological phenotypes of HOSE1-E7/hTERT (**B**), -E6/E7/hTERT (**C**) and -E6Δ151/E7/hTERT (**D**) were ‘flat-epithelial type’, while those of HOSE1s-E6/hTERT (**A**) and -E6Δ151/Bmi1/hTERT (**E**) were ‘fibroblast-like type’. Scale bar=100 *μ*m.

The growth patterns of HOSE1s cells on culture dishes were morphologically divided into two phenotypes (Figure 4 and Table 1). One was a ‘flat-epithelial type’ and was defined as a cobblestone arrangement of cuboidal epithelial-like monolayer cells, similar to the growth pattern of primary human OSE cells. The other was a ‘fibroblast-like type’ and was defined as an atypical or fusiform pattern. The growth patterns of HOSE1s-E7/hTERT, -E6/E7/hTERT, and -E6Δ151/E7/hTERT were classified as ‘flat-epithelial type’, while those of immortalised HOSE1s-E6/hTERT and -E6Δ151/Bmi1/hTERT were classified as ‘fibroblast-like type’ (Figure 4).

### Immunocytochemical characterization for cytoskeleton component

In immunocytochemistry, HOSE1s cells except for HOSE1-E6/hTERT expressed cytokeratin, vimentin, and collagen IV, but did not express EMA, similar to the primary human OSE cells (Table 3). The cells of HOSE1-E6/hTERT expressed vimentin and collagen IV, but did not express cytokeratin or EMA (Table 3).

### E-cadherin expression

The expression of E-cadherin in HOSE1s cells and an ovarian cancer cell line was examined by RT–PCR. The ovarian cancer cell line, OVCAR-3, expressed the mRNA of E-cadherin, but none of the HOSE1s cells did, identical to the primary OSE cells (data not shown).

### CA125 secretion

None of the HOSE1s cells expressed CA125 in immunocytochemistry, as with the primary OSE cells, while the ovarian cancer cell line, OVCAR-3, did (Table 1). Correspondingly, the HOSE1s cells secreted little CA125 (1.83±0.21–2.03±0.26 IU l^−1^) and OVCAR-3 highly produced CA125 (1216.67±76.38 IU l^−1^) in culture media.

### Colony formation and tumour formation in HOSE1s cells

The anchorage-independent growth property of HOSE1s cells was examined by the colony formation assay in soft agar. The colony efficiency of HOSE s-E6/hTERT, -E7/hTERT, -E6/E7/hTERT, -E6Δ151/E7/hTERT, and -E6Δ151/Bmi1/hTERT was 0.08, 0.00, 0.08, 1.55, and 0.02%, respectively. Correspondingly, the efficiency of OVCAR-3 was 49.05±5.22% (Table 1).

The cells of HOSE1-E7/hTERT and OVCAR-3 cells were injected subcutaneously into the subscapular areas of athymic *nu/nu* mice. Although tumour formation (>1 cm) was observed in four of the five mice injected with OVCAR-3 within 4 months, no tumours were found in the mice injected with HOSE1-E7/hTERT (Table 1).

## DISCUSSION

Telomere loss was previously considered to be the sole factor governing senescence since transduction of hTERT was sufficient to immortalize human fibroblasts of mesenchymal cells (Bodnar *et al*, 1998). However, other factors were also thought to be involved in senescence of human epithelial cells because several kinds of epithelial cells were unable to be immortalised by telomerase activation alone (Kiyono *et al*, 1998; Kyo *et al*, 2003). It has been generally assumed that the mortality of epithelial cells is attributed to three stages (Foster and Galloway, 1996; Foster *et al*, 1998): M0 for activation of the p16/Rb pathway, M1 for telomere shortening and activation of the p53/p21 pathway, and M2 for further shortening of telomeres in cells with inactivated p53/p21 pathway (Shay *et al*, 1991; Wright and Shay, 1992; Foster and Galloway, 1996; Foster *et al*, 1998).

OSE have the same origin as peritoneal mesothelial cells covering the intraperitoneal organs and have the properties of both epithelial and mesenchymal cells (Okamura and Katabuchi, 2005). Lately, the lifespan of human OSE cells was reported to be prolonged by telomerase activation alone, but the OSE cells were unable to be immortalised similarly to the epithelial cells (Alvero *et al*, 2004). Thereby, inhibition of the three stages, alone or in combination, was added to our experimental design for immortalisation of the mesothelial OSE cells. In particular, we transduced Bmi1 (Jacobs *et al*, 1999), E7, and E6 for inhibition of p16, Rb, and p53, respectively, as well as hTERT for activation of telomerase.

In the study presented here, five cell lines except for two lines of HOSE1s-Bmi1/hTERT and -hTERT were immortalised and grew over a population doubling level of 200. Both inhibitors of p16/Rb and p53 in combination with hTERT highly enhanced cell proliferation, and induced immortalisation. In addition, either E7 alone for p16/Rb inhibition, or E6 alone for p53 inhibition, in combination with hTERT, less enhanced it than both inhibitors. Nevertheless, their combination also successfully immortalised the OSE cells with slow cell growths.

Cell immortalisation maintaining chromosomal stability was the primary aim of the present study. After immortalisation, chromosomal analysis showed diploidy in the HOSE1-E7/hTERT cell line alone. Chromosomal alteration was revealed in the other cell lines despite hTERT-transduction to abolish chromosomal crisis. Interestingly, similar results have been reported for preadipocytes and bone marrow stromal cells immortalised by introduction of E7 and hTERT (Darimont *et al*, 2003; Mori *et al*, 2005). In these immortalised cells, functional p53 was induced by E7 probably through upregulation of p14ARF (Bates *et al*, 1998). The active p53 could have prevented chromosomally abnormal cells from emerging and proliferating. Conversely, inactivation of p53 induces chromosomal alteration by abnormal centrosome amplification (Fukasawa *et al*, 1996), and abolishes DNA damage checkpoint and p53-dependent apoptosis. The other cell lines immortalised with the help of E6 or E6Δ151 might have greater chance to accumulate chromosomal changes as a result of inactivated p53.

OSE have the properties of both epithelial and mesenchymal cells from the viewpoint of embryonal development, as described above. Cultured OSE cells have been shown to be highly responsive to environmental influences, and to exhibit mesenchymal morphology at each passage under standard culture conditions (Auersperg *et al*, 1994, 2001), that is, epithelio-mesenchymal transition (EMT). In the present study, the immortalised HOSE1s cells grew as flat epithelial cells or fibroblast-like cells. Among them, HOSE1-E7/hTERT without chromosomal instability showed flat epithelial phenotype. Immunocytochemically, the immortalised HOSE1s cells have the same cytoskeletal properties as the primary HOSE1 cells, except for cytokeratin negativity in HOSE1-E6/hTERT cells. The negativity would account EMT in cultured condition (Auersperg *et al*, 1994). However, the possibility of contamination of the stromal cells by scraping method would not be denied.

Previously, OSE cells were shown to become more firmly committed to an epithelial phenotype in the process of malignant transformation (Maines-Bandiera *et al*, 1992; Auersperg *et al*, 1994). Similar to primary human OSE cells, our immortalised HOSE1 cells did not express E-cadherin or CA125, which were expressed in the ovarian cancer cell line, OVCAR-3 (van Niekerk *et al*, 1989; Auersperg *et al*, 1999). In addition, to examine whether the immortalised HOSE1 cells were transformed, they were tested for anchorage-independent growth in a soft agar and assayed for tumorigenicity in athymic *nu/nu* mice. HOSE1-E7/hTERT, with a normal chromosomal number, did not form any colonies in soft agar medium or tumours in athymic *nu/nu* mice. Consequently, this cell line was concluded not to have major transformed phenotype.

We have been the first to establish a transduced HOSE1 cell line (HOSE1-E7/hTERT) that have no major chromosomal alterations or transformation activity. Immortalised HOSE1-E7/hTERT could now serve as a relevant model for investigation of the molecular aetiology of epithelial ovarian carcinomas, identifying markers of tumour progression. The process of ovarian carcinogenesis should also be revealed using this system.

## Figures and Tables

**Table 1:** Phenotype and growth of immortalised HOSE1s cells and ovarian cancer cell line (OVCAR-3)

	**Morphological** **type**	**Doubling** **time (h)**	**Population** **doubling level**	**Colony efficiency** **in soft agar (%)**	**Tumour formation** **in nu/nu mice**
E6/hTERT	Fibroblastic	36.9	204	0.08±0.08	N.E.
E7/hTERT	Epithelial	35.1	242	0.00±0.00	0/5
E6/E7/hTERT	Epithelial	26.1	210	0.08±0.10	N.E.
E6Δ151/E7/hTERT	Epithelial	23.4	245	1.55±0.36	N.E.
E6Δ151/Bmi1/hTERT	Fibroblastic	29.7	204	0.02±0.04	N.E.
OVCAR-3	Epithelial	N.E.	N.E.	49.05±5.22	4/5

N.E.=not examined.

**Table 2:** Chromosomal analysis of immortalised HOSE1s cells: (A) Chromosomal numbers of immortalised HOSE1s cells; (B) Karyotype analysis of immortalised HOSE1s cells

	**(A) Chromosome number**	
**Cell lines**	**Passage 16**	**Passage 41**
E6/hTERT	42–48	43–87
E7/hTERT	46–47	46
E6/E7/hTERT	43–48	43–55
E6Δ151/E7/hTERT	46–47	84–96
E6Δ151/Bmi1/hTERT	42–46	58–92
		

**Cell lines**	**(B) Karyotype analysis**	
E6/hTERT	63–87<3n>, XX, −X[10], add(X)(q22)[2], +2[2], +3[3],−4[10], add(4)(q35)[7], +5[9], +6[2], +7[8], +8[10], +8[6], +9[7], +10[7], +11[9], +12[4], add(12)(q24)[7], −13[9], −14[9], −15[8], +16[9], −17[5], +18[6], add(18)(q21)[5], +19[7], +19[2],+20[10], +20[9], +20[5], +21[10], +22[7], +22[2], +mar1[2], +mar2[3]	
E7/hTERT	A^a^ : 46XX, B^b^ : 46, XX, t(7;7)(p10;p10) add(10)(p11), C^c^ : 46, idem, −X, +i(X)(p10), add(1)(p36)	
E6/E7/hTERT	43–47, XX, ins(11;?)(q21;?)[2], −13[3], del(13)(q14)[3], −14[3], add(15)(q13)[5], ins(15;?)(q15;?)[2], +20[4], add(22)(p11)[5]	
E6Δ151/E7/hTERT	88–93<4n>, XXXX, del(X)(q2?)[3], i(1)(p10)[8], −2[10], −4[3], +5[7], +6[7], −10[4], add(10)(p11)[5], +12[9], −13[9], −13[3], −14[3], −15[10], add(15)(p11)[4], add(15)(p11)[3], −19[8], +20[10], +20[7], −22[5], +mar1[10]	
E6Δ151/Bmi1/hTERT	76–82<4n>, XXXX, del(1)(q11)x2[2], −2[9], add(3)(p11)[2], −4[7], +add(5)(p11)[2], i(5)(p10)[2], −7[4], −12[4], add(12)(p11)[3], del(12)(p11)[2], −13[10], −13[9], −15[9], −16[8], −16[4], −17[8], −17[5], −18[8], −18[3], −19[7], −22[5], +mar1[2]	

The numbers of cells were ^a^5, ^b^5, and ^c^2 of 12 cells.

**Table 3:** Immunocytochemical profile of immortalised HOSE1s cells

	**Cytokeratin**	**Vmientin**	**EMA^a^**	**Colllagen IV**	**CA125**
Primary OSE cells	+	+	−	+	−
E6/hTERT	−	+	−	+	−
E7/hTERT	+	+	−	+	−
E6/E7/hTERT	+	+	−	+	−
E6Δ151/E7/hTERT	+	+	−	+	−
E6Δ151/Bmi1/hTERT	+	+	−	+	−

a EMA=epithelial membrane antigen. +=positive, −=negative.
